# Do storybooks really break children's gender stereotypes?

**DOI:** 10.3389/fpsyg.2013.00986

**Published:** 2013-12-24

**Authors:** Carla Abad, Shannon M. Pruden

**Affiliations:** Department of Psychology, Florida International UniversityMiami, FL, USA

**Keywords:** children's storybooks, gender stereotypes, gender roles, gender *atypical* information, gender identity

## Do storybooks really break children's gender stereotypes?

Gender stereotypes—the features and characteristics assigned to men and women in a particular society—are prevalent in children as young as the preschool years (Martin and Ruble, 2004). For example, preschoolers can categorize toys as appropriate for either girls (e.g., dishset) or boys (e.g., toolset), and play with them according to gender expectations (Raag and Rackliff, 1998). Many factors have been linked to the display and development of gender stereotypes in children, including the role of storybooks in shaping children's gender stereotypes (Jennings, 1975; Ashton, 1983; Trepanier-Street et al., 1990; Green et al., 2004). Storybooks are believed to help children understand the roles of men and women in society by reinforcing children's ideas about gender roles (i.e., what is typically appropriate for men and women) or, alternatively, by *challenging* these stereotypical gender roles. But, do storybooks really break children's gender stereotypes? In addressing this question, we briefly review literature that suggests storybooks can challenge children's gender stereotypes, discuss questions left unanswered by the current literature, and set the path for future research by arguing that researchers need to examine mechanisms affecting children's interpretations of gendered information in storybooks.

## Storybooks challenge children's gender stereotypes: the evidence

### Reading gender-*atypical* storybooks increases play with gender-*atypical* toys

Children prefer to play with toys stereotypically associated with their own sex, even before the age of three (O'Brien and Huston, 1985). However, some studies suggest that gender-*atypical* storybooks (e.g., storybooks where characters display behaviors usually associated with the opposite sex) can lead to changes in children's play behavior, even in children with the most stereotypical play behavior (Ashton, 1983; Green et al., 2004). In one study, 2–5-year-olds who were read a storybook about a same-sex child engaged in play with a gender-*atypical* toy immediately showed increased play with gender-*atypical* toys (e.g., girl participant hears a story about a girl playing with a dump truck and immediately increases play with trucks; Ashton, 1983). More recently, the play behavior of eight preschoolers identified as those with the most stereotypical play behavior was observed across 4 months (Green et al., 2004). During this time, children were exposed to two storybooks in which characters displayed gender-*atypical* toy play. For some children these gender-*atypical* storybooks resulted in significant, stable changes in their play behavior, with children showing increased play with gender-*atypical* toys and decreased play with gender stereotypical toys. These studies suggest that exposure to gender-*atypical* characters and behaviors in storybooks may impact children's immediate and future play behavior.

### Reading gender-*atypical* storybooks challenges children's stereotypes about gender-appropriate occupations and activities

Young children have preconceived notions about what occupations are appropriate for males and females, and tend to select occupations stereotypically associated with their gender when asked about their future careers (Liben et al., 2001). For instance, when asked about their interest in 37 occupations, elementary school boys expressed more interest in culturally masculine jobs (e.g., professor, mechanic) than feminine jobs (e.g., gymnast, teacher), while the opposite was true for girls (Liben et al., 2001). However, children's exposure to storybooks with female protagonists in *atypical* gender roles (i.e., activities; occupations) is linked to an increase in the number of occupations children believe are appropriate for women (Scott and Feldman-Summers, 1979; Trepanier-Street and Romatowski, 1999; Karniol and Gal-Disegni, 2009). For example, 3rd- and 4th-grade children who read stories in which female protagonists were engaged in gender-*atypical* activities (e.g., story about a female explorer) were more likely to report that girls could engage in the gender-*atypical* activities portrayed in the stories (Scott and Feldman-Summers, 1979). In other research, 4–6-year-olds, upon reading and participating in teacher-mediated activities related to gender-*atypical* stories where characters were involved in *atypical* gender activities, situations, and occupations, rated more stereotypical occupations and activities as appropriate for both males and females (Trepanier-Street and Romatowski, 1999). Similarly, recent work by Karniol and Gal-Disegni (2009) found that first graders assigned gender-fair basal readers (i.e., textbooks used to teach reading) for the school year judged more activities (e.g., playing in mud, baking a cake) as appropriate for both males and females than those children assigned gender-stereotyped basal readers. These studies suggest that exposure to gender-*atypical* storybooks and readers challenge children's stereotypes about gender-appropriate occupations and activities.

### Reading gender *atypical* storybooks alters children's future goals and aspirations

Perhaps one of the most exciting findings in the storybook and gender stereotype literature is Nhundu's (2007) study of Zimbabwean girls enrolled in 4th through 7th grade. Girls exposed to biographical stories of women succeeding in non-traditional careers not only reported that there were no jobs appropriate only for men or only for women, but also reported altering their *own* future career plans from gender-*typical* to gender-*atypical*. In contrast, significantly fewer girls who had not been exposed to these gender-*atypical* biographical stories (control group) changed their career goals; girls in the control group who did alter their future career goals at the completion of the study typically reported the desire for a gender-*typical* career rather than a gender-*atypical* career. These results suggest that the reading of gender-*atypical* storybooks can alter young girls' future career goals and aspirations.

## Storybook and gender stereotype literature: unanswered questions

Despite these compelling findings that storybooks challenge children's gender stereotypes, there are some unanswered questions in the literature that should be addressed before we can assert that storybooks break children's gender stereotypes. We discuss these questions briefly, hoping they will stimulate more research on this topic.

### How are children interpreting/remembering gender-*stereotypical* and -*atypical* information?

Results are mixed with respect to how children interpret/remember gender-*atypical* information. Some find that children tend to misremember/distort gender-*atypical* information to make it consistent with gender stereotypes (Arthur and White, 1996; Daly et al., 1998; Frawley, 2008). In a study of 1st and 4th graders, children heard audiotaped readings of two books and were asked to retell stories and answer questions related to gender roles in the story (Frawley, 2008). Children who distorted gender-related information received feedback about their responses, yet they continued to misremember/reinterpret gender-*atypical* information to make it consistent with gender stereotypes. Still, others suggest that children remember gender-*atypical* information *better* due to novelty effects (Jennings, 1975; Trepanier-Street and Kropp, 1987). In another study, 32 preschool-aged children were read two different stories in which the character of the same sex: (1) performed a gender stereotypical activity (e.g., girl wanted to be a ballerina); and (2) performed a gender-*atypical* activity (e.g., boy wanted to be a dancer). After hearing these two stories, children were asked to report which story they preferred and were tested on their recall of information from each story. While children preferred stories that were consistent with gender stereotypes, they tended to remember information from the gender-*atypical* stories better. Given these mixed results from the literature, we argue that additional research be conducted to identify those factors (e.g., child age, child sex, child's previously-held stereotypes, amount of exposure to storybooks, and storybook plot/characters) that moderate children's memory for gender-*stereotypical* and –*atypical* information from storybooks.

### Do storybooks challenge gender stereotypes in all children?

The child's sex (Daly et al., 1998) and previously held stereotypes (Green et al., 2004) affect how responsive the child is to gender-*atypical* information in storybooks. Some find that the play behavior of some children does not change after exposure to gender-*atypical* storybooks, particularly in those children who are rated as having robust gender stereotypes (Green et al., 2004). Others argue that sex of the child is a good predictor of whether exposure to gender-*atypical* information in storybooks will impact the child. In many studies, girls' but not boys' gender stereotypes and play behavior can be altered with exposure to gender-*atypical* storybooks (e.g., Daly et al., 1998; Green et al., 2004). While both boys and girls can recall storybook information well when the protagonist is of the same sex and behaves in gender stereotypical ways, girls tend to remember more information about the story than boys when the protagonist does not conform to gender stereotypes (Daly et al., 1998). Others find that boys show gender stereotypes at a younger age and are less flexible in modifying these stereotypes (Ashton, 1983). Additionally, although it has been suggested that young children (5–6-year-olds) have the most rigid gender stereotypes (Ruble et al., 1998; Trautner et al., 2005), there is little conclusive evidence for differences in responsiveness to gender-*atypical* information in storybooks based on child age. Thus, more research is needed to evaluate potential sex and age differences on children's ability to recall gender-*atypical* information from storybooks, as well as whether and how boys' gender stereotypes can be altered using storybooks.

### How much and what kind of exposure to gender-*atypical* information in storybooks is needed to promote lasting change?

Studies evaluating the effectiveness of gender-*atypical* storybooks in changing children's gender stereotypes vary considerably in the kind and amount of exposure to these stories. Some studies have looked at children's behavior and stereotypes after just one exposure to a gender-*atypical* storybook (Ashton, 1983). Others have examined the effects of exposure to six different gender-*atypical* storybooks, along with teacher-mediated activities related to those storybooks, across 2 months (Trepanier-Street and Romatowski, 1999). Nhundu (2007) examined participants after a year of exposure to storybooks. Most published works assess only *immediate* change after exposure and find mixed results [e.g., Flerx et al. (1976) found an initial reduction of stereotypical thinking in preschoolers was not evident at 1-week follow-up]; to our knowledge, no studies have evaluated whether exposure results in long-lasting and/or permanent change in children's gender stereotypical beliefs/behaviors.

### How generalizable is the gender-*atypical* information in storybooks?

It is unclear whether information from gender-*atypical* storybooks extends beyond the specific circumstance the story portrays (i.e., activities, toys, careers). Participants are more likely to report that girls can engage in gender-*atypical* activities and play with gender-*atypical* toys portrayed in a storybook (Scott and Feldman-Summers, 1979), but do these beliefs/behaviors extend beyond the particulars of the storybook? Although some suggest generalizability (Trepanier-Street and Romatowski, 1999), there is reason to believe that these beliefs/behaviors do not extend beyond specific storybook content. While Nhundu (2007) saw that over 73% of girl participants had changed their career goals from traditional to non-traditional careers after long-term exposure to gender-*atypical* storybooks, she also reported that participants still held *traditional* gender role beliefs, such as that there are tasks only for men or women within the home setting and that married women should not have jobs that keep them from their in-home responsibilities. These results suggest that while storybooks have the potential to change children's gender role beliefs/behaviors, rather than challenging children's gender stereotypes in general, these changes are limited to the specific beliefs/behaviors portrayed in the storybooks.

## Storybook and gender stereotype research: a call for further research

Storybooks are one way children learn about the world, learn about gender stereotypes, and potentially break gender stereotypes. Children's storybooks can provide a representation of societal values during a time when children are developing their gender roles, and thus, represent an important avenue for research on children's developing gender stereotypes. Gender-*atypical* storybooks may be a relatively easy, inexpensive way to expose children to a more gender-equitable worldview at home or in the classroom and, may serve as a powerful tool to challenge gender stereotypes. Existing evidence begins to shed light on how storybooks break gender stereotypes with studies documenting that reading gender-*atypical* storybooks not only increases play with gender-*atypical* toys and challenges children's stereotypes about gender-appropriate roles, but also alters children's future career goals and aspirations. There are, however, many questions to which we do not have an answer, including those that we addressed in the previous section. To understand the true impact storybooks have on children's developing gender stereotypes, we must examine the potential mechanisms explaining children's changing preferences from *typical* to *atypical* gender roles, beliefs, activities, and toys. We have identified several factors ripe for investigation and a careful examination of these potential factors will finally allow us to answer the question, “do storybooks really break children's gender stereotypes?”
